# Stress analysis of parallel oil and gas steel pipelines in inclined tunnels

**DOI:** 10.1186/s40064-015-1453-1

**Published:** 2015-10-31

**Authors:** Xiaonan Wu, Hongfang Lu, Shijuan Wu

**Affiliations:** State Key Laboratory of Oil and Gas Reservoir Geology and Exploitation, Southwest Petroleum University, Chengdu, Sichuan China; School of Civil Engineering and Architecture, Southwest Petroleum University, Chengdu, Sichuan China; School of Petroleum Engineering, Southwest Petroleum University, Chengdu, Sichuan China

**Keywords:** Stress analysis, Parallel oil and gas pipelines, Inclined tunnel, Displacement analysis, Earthquake action, Stress influencing factors

## Abstract

Geological conditions along long distance pipelines are complex. In consideration of differences in elevation and terrain obstacles, long distance pipelines are commonly laid through tunnels. Oil and gas pipelines are often laid side by side to reduce construction costs and minimize geological impact. The layout and construction of parallel oil and gas pipelines are more complex than those of single pipelines. In order to reduce safety hazards, it is necessary to carry out stress analysis of the oil and gas pipelines that run through tunnels. In this study, a stress analysis model of pipelines running through a tunnel was developed. On the basis of the finite element method, CAESAR II software was used to analyze the stress and displacement of a section of parallel oil and gas pipelines that run through tunnels and stress and displacement distribution laws were drawn from the analyses. A study of the factors influencing stress recommended that: (1) The buttress interval of the parallel oil and gas pipelines in a tunnel should be 12 m; (2) The angle of inclined pipelines should be no greater than 25°; (3) The stress of oil pipelines enhances more obviously than that of gas pipelines under earthquake action; (4) The average stress can be reduced by adopting “ladder” laying; and (5) Guide bend can be set at the tunnel entrance and exit in order to reduce the stress.

## Introduction and background

Long distance oil and gas pipelines share complex external environments, and terrain restrictions and maintenance negligence can compound the likelihood and magnitude of accidents. Pipelines are laid through tunnels mainly by directional drilling, shields, and pipe jacking to overcome elevation and terrain obstacles, facilitate pipeline construction, and minimize the destruction of surface vegetation and soil erosion. This approach can also reduce construction and pipeline maintenance costs.

Oil and gas pipelines can fail in tunnels for a number of reasons. In addition to design errors, the quality of construction, pipeline corrosion and fatigue, and insufficient strength in the bends of pipelines can all contribute to pipeline failure. Therefore, it is vital to carry out stress analysis of pipelines in comparable settings before construction begins.

In the 1930s and 40’s, different methods in structural mechanics were used to combat the internal forces in piping systems (Watkins and Anderson 1999). One of the first methods was the elastic center method, which was well-developed and simple (Sokolnikoff and Specht 1956; Yu and Lv 2008). However, it resulted in great errors when used in calculations involving inclined pipelines or a large number of arc elements. Later, basic methods for solving statically indeterminate structures were used (Liu and Ando 2000), which included written calculations and a foundation for computing matrices that would eventually be accomplished with electronic computers. In the 1950s, people began to use the matrix method of structural analysis for calculations of pipelines to solve the forces, moments, and displacements at the ends of pipeline systems (Zhang 1993; Peng 1978). Karamitros et al. (2007) proposed a stress analysis model for strike-slip fault. Although common assumptions were used in the model, a series of improvements were introduced, making the model more extensively applicable. In 2009, *Pipe Stress Engineering*, written by a structural engineer named Liang-Chuan Peng, was published (Peng and Peng 2009), and pipeline stress analysis system was perfected. Currently, stress analysis of pipelines can be carried out using the finite element method with the help of sophisticated software such as CAESAR II and ANSYS. Wu et al. (2012) analyzed the static stress of a gas pipeline running through an inclined tunnel, she came to the tunnel gas pipeline stress concentration point, and determined that the pressure is the main factor influencing the stress of tunnel pipeline. However, she didn’t do deep analysis on other influencing factors. Vazouras et al. (2010) did research on buried steel pipeline crossing strike-slip fault, using ABAQUS to simulate the interaction between pipe and soil based on shell model. Pike et al. (2010) did research on submarine pipeline buckling using ABAQUS under the effect of high temperature and high pressure. Xiong et al. (2013) simulated the dynamic response of a buried pipeline induced by a rock-fall impaction using finite element software.

From recent stress analysis research, it can be seen: (1) there is few stress study on tunnel pipeline. (2) study on tunnel pipeline mainly focuses on gas pipeline. However, parallel pipelines (gas pipeline and oil pipeline) are much more common, but there are few researches on tunnel pipeline stress, resulting in the lack of comprehensive consideration of tunnel pipeline design. (3) Previous study of the tunnel pipeline was confined to conventional working condition. (4) Pipeline stress analysis technology is in the development of the static to dynamic, while present dynamic study was confined in ordinary buried pipelines, and there is little study on tunnel pipeline. (5) The difference between tunnel pipeline and buried pipeline is that displacement check for tunnel pipeline is as important as meeting stress requirements. However, the previous research did few analyses on pipeline displacement.

The particularity of parallel oil–gas pipelines is taking stress of both pipelines into account simultaneously. In other words, you have to redesign when one of them under certain circumstances satisfies the requirement of stress while the other one does not satisfy. Parallel pipeline is studied in this paper, and the significance is to provide the basis for the design, to shorten the design cycle, and to avoid the duplication design. This paper established a universal model for pipelines laid in tunnels based on engineering practice, using CAESAR II software to conduct stress and displacement analyses of a certain section of the parallel oil and gas pipelines. Then, the factors influencing stress, such as the length of the inclined pipelines, the angle of the inclined pipelines, the buttress interval, the earthquake action, and stress reducing measures were analyzed to provide a basis for pipeline design in engineering applications.

## Case study

### Tunnel structure

**Fig. 1:**
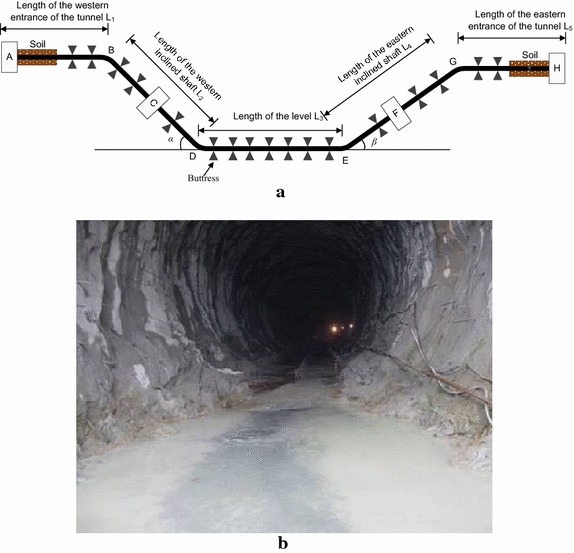
Schematic of a typical tunnel structure model: **a** physical model; and **b** actual project

The basic form of a tunnel-laid pipeline follows the “inclined shaft-level-inclined shaft” structure, and can be divided into two parts: the pipeline in the tunnel and the pipeline outside the tunnel. The pipe piers and lines in a typical tunnel are laid out as shown in Fig. 1, where fixed piers A and H are installed on both sides of the model. The pipeline at the entrance (L_1_) and exit (L_5_) of a tunnel is generally laid horizontally and covered by soil, and no buttresses are used. L_2_ is the length of the western inclined shaft, with an angle of *α*, and anchor block C is installed in the middle. L_4_ is the length of the eastern inclined shaft, with an angle of *β*, and anchor block F is installed at a distance from bend G.

**Fig. 2:**
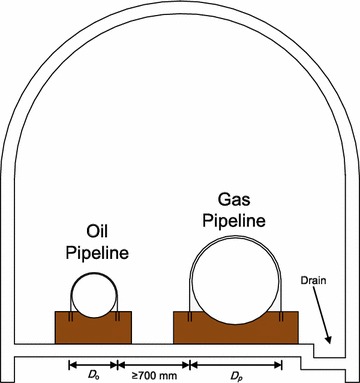
Cross-section diagram of the parallel oil and gas pipelines

Parallel oil and gas pipelines in a tunnel are generally supported at intervals by buttresses. The structural parameters of the two pipelines (the length of the inclined pipelines, the angle of the inclined pipelines, and the lengths of the inclined and horizontal sections) are the same. A cross-section diagram of the parallel oil and gas pipelines is shown in Fig. 2, where *D*_*o*_ represents the diameter of the oil pipeline and *D*_*p*_ represents the diameter of the gas pipeline.

### Stress analysis method

#### Pipeline model

There are typically two models for stress analysis of pipelines: the beam model and the shell model. The shell model is suitable for the local analysis of pipelines, and the beam model is typically used for the stress analysis of long-distance pipelines (Jiang et al. 2013). A three-dimensional beam element model has six degrees of freedom (Beam element has 2 nodes, each node has 3 degrees of freedom, 6 degrees of freedom include 3 translational degrees of freedom and 3 rotational freedom) (Sreejith et al. 2004).

In addition to straight sections, a long-distance pipeline also has bends that allow it to change its course. The beam element model is used for bends as well as straight sections; the difference is that flattening occurs in the section of the pipeline in the direction of the bend radius. Therefore, the concept of “stress intensification factor” is used to describe the effect of stress concentration at a bend. Relevant parameters, such as the stress intensification factor, can be obtained from Appendix D in ASME B31.3 (2012a).

#### Grid generation

Currently, CAESAR II software is widely used to research stress analysis of pipelines and has been validated through projects with high analysis precision and reliable analysis results. The gravity of a pipeline is equally divided between the nodes at both ends in CAESAR II. If a pipe section is too long, and the gravity divided between the nodes at both ends is too large, stress may fail to pass a test. In order to ensure the reasonableness and conciseness of the analyses, we often use *n*_s_ = (0.5–5) *D* for a pipeline, where *n*_s_ represent the distance between the nodes and *D* represents the diameter of a pipeline (Jiang et al. 2013; George 1998). Actual node distance should be chosen according to the length of pipeline, the smaller the distance, the more accurate the calculation results. We often choose *n*_s_ = (0.5–1) *D* when the pipe is not long.

#### Stresses of pipelines

In Cartesian coordinate system, In Cartesian coordinate system, one micro-element hexahedron from the pipeline, on which there are 9 stress component in total, is taken as example. According to theorem of conjugate shearing stress, only six components of the nine are independent: $\sigma_{x}$, $\sigma_{y}$, $\sigma_{z}$, $\tau_{xy}$, $\tau_{xz}$ and $\tau_{yz}$. So once the six stress components are known, the stress of that point in an arbitrary direction can be calculated. In the pipeline, based on the direction of stress, pipeline stress can be classified into axial stress, hoop stress, radial stress and shear stress. And according to the failure mode of pipeline, pipe stress can also be divided into primary stress, secondary stress and peak stress. The difference among these stress lies in their diverse load. However, while checking the stress, it is quiet complex to singly verify a stress in a certain direction. So according to different working conditions, namely different loads, the concept of code stress is introduced: if the code stress does not exceed the allowable value of stress, then no damage will happen since the stress under that working condition meets the demand. Equation (1)–(3) are the calculation formulas of code stress under peak stress, primary stress and secondary stress calculation conditions respectively (Song 2011).

- Code stress of peak stress calculation condition:
  1 $$\overrightarrow {{\sigma_{cs} }} = \frac{{\overrightarrow {{F_{ax} }} }}{A} + \frac{{\overrightarrow {P} D}}{4t} + \frac{{\overrightarrow {M} }}{W}$$
- Code stress of primary stress calculation condition:
  2 $$\overrightarrow {{\sigma_{cs} }} = \frac{{\overrightarrow {P} D}}{4t} + \frac{{\overrightarrow {M} }}{W}$$
- Code stress of secondary stress calculation condition:
  3 $$\overrightarrow {{\sigma_{cs} }} = \frac{{\overrightarrow {{F_{ax} }} }}{A}$$

where $\overrightarrow {{\sigma_{cs} }}$ is code stress, MPa; $\overrightarrow {{F_{ax} }}$ is axial force which is not caused by pressure, N; $A$ is cross-sectional area, mm^2^; $\overrightarrow {P}$ is pipeline pressure, MPa; $D$ is pipeline diameter, mm; $t$ is thickness, mm; $M$ is resultant bending moment, N mm; $W$ is module of bending section, mm^3^.

#### Pipeline beam element properties

Pipeline beam element has three major features: (1) Obeying Hooke’s law, the main deformation characteristic is bend; (2) The mechanical behavior of every element is described by end point, including thrust, displacement and stress; (3) the calculation of pipe analysis model constructed by beam element requires the basic material parameters, including stiffness, diameter, thickness, length, elasticity modulus, Poission’s ratio, linear expansion coefficient and density.

The mechanical properties hypothesis of beam element is:

- Ignore local deformation;
- Warping does not exist in any cross section of pipelines, namely assuming the pipeline follows pure bending deformation;
- Ignore the collision impact between pipes;
- Shear force is not the focus of the research;
- Supporting function is applied on unit center line.

#### Standards for stress, strain and displacement of pipelines

##### Checking stress

Pipelines in tunnels that are not embedded in soil should still comply with ASME B31.8 (2012b) *Gas Transportation and Distribution Piping Systems*, while stress-checking of crude oil pipelines should comply with ASME B31.4 (2012c) *Pipeline Transportation Systems for Liquids and Slurries*.

According to the provisions of paragraph 833.6 in ASME B31.8 and paragraph 403.3 in ASME B31.4, stress-checking should be performed on gas and oil pipelines, as shown in Table 1. (Note: $\sigma_{H}$ represents primary stress, $\sigma_{E}$ represents secondary stress, $\sigma_{L}$ represents peak stress, and $\sigma_{s}$ represents pipeline’s minimum yield stress).

**Table 1:** Stress-checking requirement of oil and gas pipelines

Stress type	Gas pipeline	Oil pipeline
Peak stress	$\sigma_{L} \le 0.90\sigma_{s}$	$\sigma_{L} \le 0.90\sigma_{s}$
Primary stress	$\sigma_{H} \le 0.75\sigma_{s}$	$\sigma_{H} \le 0.72\sigma_{s}$
Secondary stress	$\sigma_{E} \le 0.72\sigma_{s}$	$\sigma_{E} \le 0.90\sigma_{s}$

##### Checking strain

Tensile strain should meet the following requirement:

4 $$\varepsilon_{tf} \le n\phi_{\varepsilon t} \varepsilon_{t}^{crit}$$

where *ε*_*tf*_ is factorization tensile strain, dimensionless, %; *n* is design factor, dimensionless. 0.72 for oil pipeline. For gas pipeline, we need to take location classes into account (0.72 for first class area, 0.6 for second class area, 0.5 for third class area, and 0.4 for fourth class area); $\phi_{\varepsilon t}$ is weld stiffness coefficient, usually take 0.7; $\varepsilon_{t}^{crit}$ is pipeline allowable strain, usually take 0.75 %.

Compressive strain should meet the following requirement:Earthquake action case

5 $$\varepsilon_{cf} \le \varphi_{\varepsilon c} \varepsilon_{c}^{crit}$$

where *ε*_*cf*_ is factorization longitudinal or circumferential compressive strain, dimensionless; *φ*_*εc*_ is compressive strain damping factor, dimensionless, usually take 0.8; $\varepsilon_{c}^{crit}$ is longitudinal or circumferential limit compression strain, dimensionless, usually take values by Eq. (6).

6 $$\varepsilon_{c}^{crit} = \left\{ \begin{aligned} 0.5\frac{t}{D} - 0.0025 + 3000\left( {\frac{{\left( {p_{i} - p_{e} } \right)D}}{2tE}} \right)^{2} ,\frac{{\left( {p_{i} - p_{e} } \right)D}}{{2t\sigma_{s} }} < 0.4 \hfill \\ 0.5\frac{t}{D} - 0.0025 + 3000\left( {\frac{{0.4\sigma_{s} }}{E}} \right)^{2} ,\frac{{\left( {p_{i} - p_{e} } \right)D}}{{2t\sigma_{s} }} \ge 0.4 \hfill \\ \end{aligned} \right.$$

where *t* is thickness of pipeline, mm; *D* is pipeline diameter, mm; *p*_*i*_ is maximum design pressure, MPa; *p*_*e*_ is minimum external hydrostatic pressure, MPa; *E* is elasticity modulus, MPa; *σ*_*s*_ is pipeline’s minimum yield stress, MPa.

##### Checking displacement

According to GB 50316 (2008), displacement checking primarily verifies whether the following conditions are met:

- The angular displacement of a horizontal pipeline is generally required to be no greater than 4°.
- The linear displacement of a horizontal pipeline should not exceed 40 % of the length of a sliding pipe bracket.

### Displacement, stiffness and mass matrix of pipeline in finite element method

According to Tang Yongjin’s *Pressure Piping Stress Analysis* (Tang 2003), in order to study the overall equilibrium of a pipeline system, an element matrix needs to be expanded in order to be equivalent to a pipeline matrix (Pipeline matrix includes stiffness matrix and mass matrix, which respectively indicate elastic properties and inertia properties) (Xiao 2006). If there are *n* nodes in a pipeline system, the pipeline system has 3*n* node displacements (active degrees of freedom).

#### Stiffness matrix

7 $$[K] = \sum\limits_{e = 1}^{m} {[G]^{T} [\overline{{K^{e} }} ][G]}$$

where [*K*] is the stiffness matrix of an overall pipeline system; [*G*] is the transformation matrix from element nodes to pipeline system nodes; $[\overline{{K^{e} }} ]$ is the element stiffness matrix in a global coordinate system.

#### Mass matrix

8 $$[M] = \sum\limits_{e = 1}^{m} {[G]^{T} [\overline{{M^{e} }} ][G]}$$

where [*M*] is the mass matrix of an overall pipeline system; [*G*] is the transformation matrix from elements nodes to pipeline system nodes; $[\overline{{M^{e} }} ]$ is the element mass matrix in a global coordinate system.

### Constraints

#### Buttress (Pipe clamp)

**Fig. 3:**
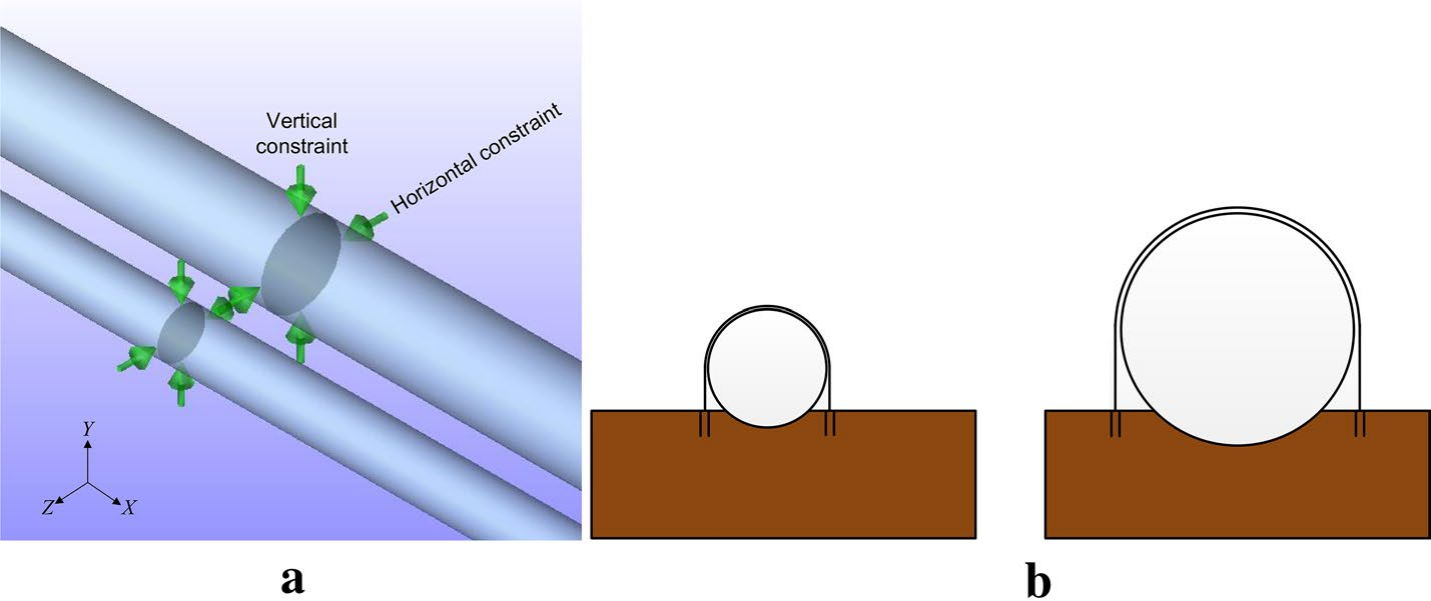
Schematic of buttress (pipe clamp) constraint: **a** software simulation results; and **b** schematic of the actual constraint

Most pipelines inside tunnels are supported by buttresses, and only a few pipelines inside tunnels are actually buried in soil. The buttresses are typically equipped with pipe clamps that can limit the axial, vertical and horizontal displacements of pipeline to some degree (Allow some movements) (Fig. 3). The constraint conditions are shown in Eq. (9).

9 $$\left\{ \begin{aligned} &dy \ge 0 \hfill \\ &dz \ge 0 \hfill \\& f = \mu_{1} \left[ {\pi D\rho_{p} gt + 0.25\pi g\left( {D - t} \right)^{2} \rho_{f} } \right] + \mu_{2} W_{2} \hfill \\ \end{aligned} \right.$$

where *dy* is the vertical (orthogonal to the pipe axis) displacement of a pipeline; *dz* is the horizontal displacement of a pipeline; *D* is the outside diameter of a pipeline, measured in m; *t* is the wall thickness of a pipeline, measured in m; *f* is the friction per unit length of a pipeline, measured in N/m; *ρ*_*p*_ is the density of a pipeline, measured in kg/m^3^; *ρ*_*f*_ is the density of the fluid in a pipeline, measured in kg/m^3^; *μ*_1_ is the friction coefficient between the pipeline wall and the pipeline support; *μ*_2_ is the friction coefficient between the pipeline wall and the pipe clamp; and *W*_2_ is the load caused by thermal stress.

#### Anchor block

An anchor block is typically located in the middle of an inclined pipe. It constrains the vertical and horizontal displacements of a pipe as well as its axial displacement. In CAESAR II, the constraints in the Z direction (horizontal), Y direction (vertical), and LIM (axial limit) are used.

#### Soil

Soil constrains the movement of the pipeline in the axial, horizontal, and vertical directions. In actual conditions, the curve describing the relationship between soil deformation and constraints is nonlinear, but usually linear processing is adopted. For simplicity of analysis, soil constraints can be considered linear constraints. Continuous soil is typically discretized into three one-way springs with bilinear stiffness. The stiffness of a soil spring is the slope of its actual deformation-constraint curve and is usually solved using the Peng model (Peng 1978).

### Boundary conditions

#### Boundary conditions of fixed piers

In order to prevent bending caused by the weight of the entire pipeline system, fixed piers are installed to eliminate the effects of the pipeline outside the tunnel on the pipeline inside the tunnel. Fixed piers are constrained from displacing and bearing axial forces, but they can bear bending moments and shear forces (Jiang et al. 2013).

#### Boundary conditions of overlying soil

In the model, only a small section of the pipeline was covered by soil on either side of the pipeline in the tunnel, and there were no bends. Therefore, the boundary conditions of both ends can be simplified to axial constraints (Jiang et al. 2013).

### Project profile

**Fig. 4:**
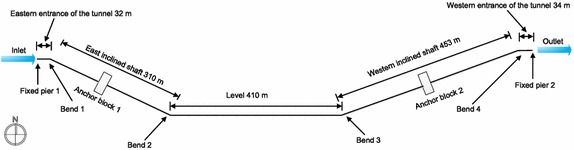
Schematic of the section of the parallel oil and gas pipelines in the tunnel

According to the design data for a particular section of oil and gas pipelines (Fig. 4), the total distance between the start and end points of the pipeline was 1240 m and the pipeline in the tunnel was 1175 m long, where it was exposed and supported by pipe racks. At the entrance and exit of the tunnel, Fixed Pier 1 and Fixed Pier 2 were installed, respectively. The entrance of the tunnel was 32 m long and a fixed pier was installed. The western inclined shaft was 310 m, with an angle of 25°, 16 buttresses at 18 m intervals, and an anchor block (installed in the middle of the western inclined shaft). The level portion of the pipeline was 410 m and included 22 buttresses. The eastern inclined shaft was 455 m, with an angle of 20°, 24 buttresses, and an anchor block (positioned at the center of the eastern inclined shaft). The exit portion of the tunnel was 34 m, with one fixed pier. The buttresses of the eastern inclined shaft were installed at 12 m intervals, and the other buttresses were installed at 18 m intervals. The pipe brackets of the buttresses were 1.5 m. The radius of curvature of the hot-fabricated bends of the gas pipeline was R = 6*D*, and the radius of curvature of the bends of the crude oil pipeline was R = 10*D* (*D* represents the outside diameter of the pipeline).

Two pipelines were required for numerical simulation. The distance between the two pipelines was 700 mm. The gas pipeline was made of API X80 longitudinally-submerged arc welded steel pipe with a diameter of 1016 mm. Its operating temperature was 50 °C with an operating pressure of 10 MPa. The crude oil pipeline was also made of API X80, with a diameter of 610 mm. Its operating temperature was 20 °C with an operating pressure of 9 MPa. Concrete parameters of oil and gas pipelines are shown in Table 2, and soil parameters are shown in Table 3 (L represents length in Thermal expansion coefficient, the unit can be unified). Material constant for X80 steel pipeline: elasticity modulus E = 206GPa, Poisson’s ratio *μ* = 0.3, density *ρ* = 7850 kg/m^3^.

**Table 2:** Oil and gas pipelines’ parameters

Pipelines	Diameter (mm)	Thickness of straight pipe (mm)	Thickness of pipe bend (mm)	Corrosion (mm)	Pressure (MPa)	Temperature (°C)	Fluid density (kg/m^3^)	Minimum yield stress (MPa)
Gas	1016	18.4	22.2	1	10	50	95	551
Oil	610	7.9	7.9	1	9	20	900	551

**Table 3:** Soil parameters

Friction coefficient	Soil density (kg/m^3^)	Buried depth to top of pipe (m)	Friction angle (degree)	Yield displacement factor	Overburden compaction multiplier	Thermal expansion coefficient (L/L/ °C)
0.4	2400	1.20	30	0.015	5	11.214 × 10^−6^

### Numerical simulation

There are three steps to establish the numerical model in CAESAR II software (Lu et al. 2015): (1) Establish basic model, (2) Input constraints and (3) Establish loading conditions.

#### Establish basic model

A pipeline model was established according to the actual strike of the pipeline and mainly consisted of straight pipes and bends. In this section, we need to input some values about pipeline such as diameter, thickness, temperature, pressure and some pipeline material parameters.

#### Input constraints

According to the actual conditions of the pipeline, constraints were simplified and loaded to the pipelines.

#### Loading conditions

The loads applied to pipelines in production and operating conditions differ. Therefore, on the basis of analytical requirements, different operating conditions were established. In order to analyze whether the primary stress, secondary stress, and peak stress of the pipelines met the standards, varying operating conditions were established in the CAESAR II software according to the characteristics of the different types of stress (Wu et al. 2014, 2015). The operating conditions and their respective stresses are shown in Table 4, where W represents gravity, P represents pressure, T represents temperature, F represents impact, WW represents the gravity of the pipeline after being filled with water and HP represents the hydrotest pressure.

**Table 4:** Load cases

Loading conditions	Representation in CAESAR II	Stress type	Remark
Operating case of gas pipeline	W + P1 + T1	Peak stress	P1 = 10 MPa, T1 = 50 °C
Operating case of oil pipeline	W + P2 + T2	Peak stress	P2 = 9 MPa, T2 = 20 °C
Sustained case of gas pipeline	W + P1	Primary stress	P1 = 10 MPa
Sustained case of oil pipeline	W + P2	Primary stress	P2 = 9 MPa
Expansion case of gas pipeline	T1	Secondary stress	T1 = 50 °C
Expansion case of oil pipeline	T2	Secondary stress	T2 = 20 °C
Pigging case of gas pipeline	W + P1 + T1 + F1	Peak stress	P1 = 10 MPa, T1 = 50 °C
Pigging case of oil pipeline	W + P2 + T2 + F2	Peak stress	P2 = 9 MPa, T2 = 20 °C
Pressure test case of gas pipeline	WW + T3 + HP	Peak stress	T3 = 15 °C, HP = 15 MPa
Pressure test case of oil pipeline	WW + T4 + HP	Peak stress	T4 = 15 °C, HP = 13.5 MPa
Earthquake action case	W + P+ T + U_*i*_	Peak stress	U represents earthquake acceleration, *i* represents the direction of earthquake action

It should be pointed out that during pigging, the velocity of the spherical pig in the gas pipeline was 5 m/s and 3.5 m/s in the oil pipeline. Hydrostatic pressure testing was used for both the gas and oil pipelines in which the test pressure was 1.5 times the design pressure and the test temperature was 15 °C.

## Results

There has been no researcher discussed on the displacement of the pipeline in tunnel, nor to contrast the results of the pipeline in tunnel under various conditions, especially the pigging condition. In this paper, stress and displacement of oil and gas pipelines under operation, test pressure and pigging conditions were studied.

### Stress of pipelines

**Fig. 5:**
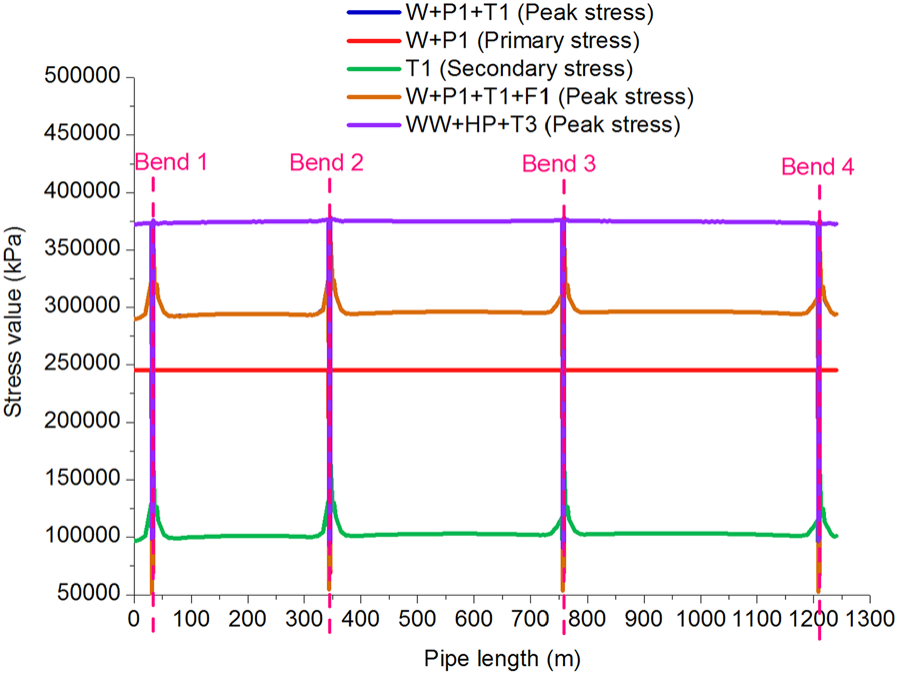
Stress distribution of the gas pipeline

**Fig. 6:**
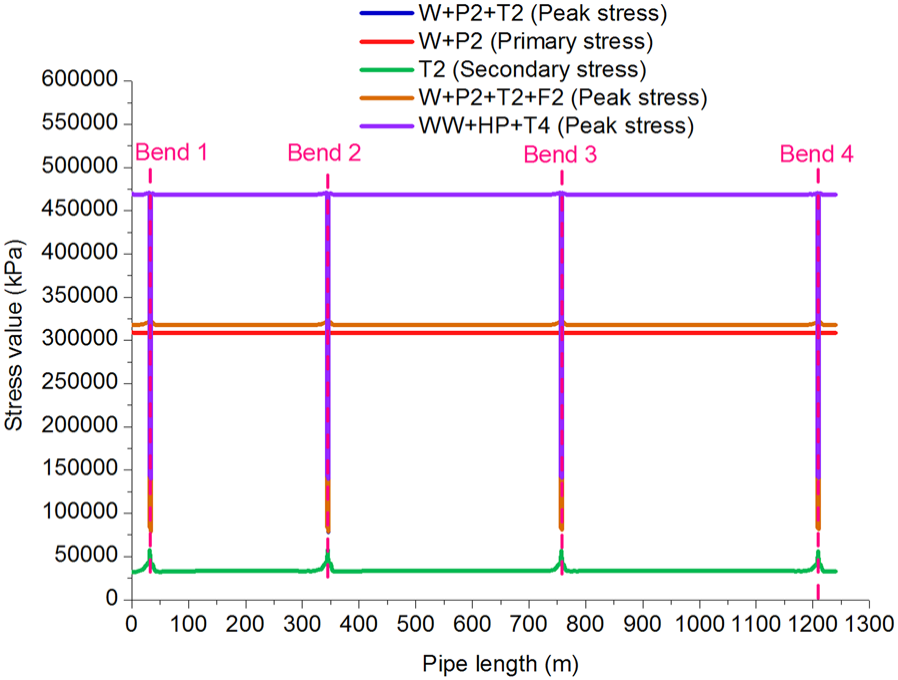
Stress distribution of the oil pipeline

Figures 5 and 6 illustrate the stress distributions of the gas pipeline and the crude oil pipeline in different loading conditions. Tables 5 and 6 show the checking of the various types of stress on the gas and oil pipelines. The following conditions were observed:

**Table 5:** Stress-checking of the gas pipeline

Loading conditions	Representation in CAESAR II	Stress type	Maximum stress value (MPa)	Location	Average stress value (MPa)	Stress check value (MPa)
Operating case of gas pipeline	W + P1 + T1	Peak stress	362.21	Bend 2	285.57	551 × 0.9 = 495.90
Sustained case of gas pipeline	W + P1	Primary stress	245.39	Fixed pier 1	234.70	551 × 0.75 = 413.25
Expansion case of gas pipeline	T1	Secondary stress	169.58	Bend 2	107.36	551 × 0.72 = 396.72
Pigging case of gas pipeline	W + P1 + T1 + F1	Peak stress	362.71	Bend 2	285.57	551 × 0.9 = 495.90
Pressure test case of gas pipeline	WW + T3 + HP	Peak stress	378.45	Bend 3	358.03	551 × 0.9 = 495.90

**Table 6:** Stress-checking of the crude oil pipeline

Loading conditions	Representation in CAESAR II	Stress type	Maximum stress value (MPa)	Location	Average stress value (MPa)	Stress check value (MPa)
Operating case of oil pipeline	W + P2 + T2	Peak stress	321.75	Bend 3	305.85	551 × 0.9 = 495.90
Sustained case of oil pipeline	W + P2	Primary stress	309.13	Fixed pier 1	297.78	551 × 0.75 = 413.25
Expansion case of oil pipeline	T2	Secondary stress	57.96	Bend 2	34.94	551 × 0.72 = 396.72
Pigging case of oil pipeline	W + P2 + T2 + F2	Peak stress	321.76	Bend 3	305.86	551 × 0.9 = 495.90
Pressure test case of oil pipeline	WW + T4 + HP	Peak stress	470.70	Bend 3	451.39	551 × 0.9 = 495.90

1. The stress in the gas and oil pipelines in the different cases did not exceed the permitted stress values, meeting ASME B31.8 and ASME B31.4 requirements.
2. The average stress in the gas and oil pipelines was the highest in the pressure test and the lowest in the expansion case. Therefore, the pressure test can be defined as a dangerous test in oil and gas pipelines, and during design, focus should be placed on diligently checking pipeline stress during the pressure test.
3. The impact of pigging had a small effect on the stress in the pipelines. Pigging increased the stress in the gas pipeline by 0.14 % and by 0.003 % in the oil pipeline, indicating that pigging has relatively significant effects on gas pipelines relative to oil pipeline. Because the angles of the inclined pipelines in this study were small, the effects of pigging were not significant. In high and steep slope projects where the angle is extreme, importance should be attached to the stress analysis of gas pipelines during pigging considering the great compressibility of gas.
4. The maximum stress in the gas and oil pipelines in the various cases occurred at Fixed Pier 1, Bend 2, and Bend 3. Therefore, these locations can be defined as dangerous sections of the pipelines. In addition, the maximum stress in all the other cases, in addition to the sustained case, occurred at Bend 2 and Bend 3, which was caused by the uneven stress distributions due to the lack of supports at the bends and the sudden changes in the course of the pipelines, as well as by the greater effects of the gravity of the fluid in the pipelines on Bend 2 and Bend 3 in comparison to Bend 1 and Bend 4.
5. The fluid used in the gas and oil pipeline pressure testing was water at a temperature of 15 °C. The hydrotest pressure in the gas pipeline (15 MPa) was greater than that in the oil pipeline (13.5 MPa), but the average stress in the gas pipeline (358.03 MPa) was smaller than that in the oil pipeline (451.39 MPa), showing that a greater pipe diameter results in a superior ability to bear pressure.

### Strain of pipelines

According to Eq. (4), for gas pipeline, *f* = 0.72, $\phi_{\varepsilon t}$ = 0.7, $\varepsilon_{t}^{crit}$ = 0.5 %, we obtained tensile strain *ε*_*tf*_ should be less than 0.378 %. According to Eq. (5)–(6), *φ*_*εc*_ = 0.8, *t* = 18.4, *D* = 1016, *p*_*i*_ = 10, *p*_*e*_ ≈ 0, *E* = 2.06 × 10^5^, *σ*_*s*_ = 551,we obtained compressive strain $\varepsilon_{c}^{crit}$ should be less than 0.799 %. According to the simulation results, it indicates that the maximum strain 0.183 % of this gas pipeline does not exceed the allowable value.

According to Eq. (4), for oil pipeline, *f* = 0.72, $\phi_{\varepsilon t}$ = 0.7, $\varepsilon_{t}^{crit}$ = 0.5 %, we obtained tensile strain *ε*_*tf*_ should be less than 0.378 %. According to Eq. (5)–(6), *φ*_*εc*_ = 0.8, *t* = 7.9, *D* = 610, *p*_*i*_ = 9, *p*_*e*_ ≈ 0, *E* = 2.06 × 10^5^, *σ*_*s*_ = 551, we obtained compressive strain $\varepsilon_{c}^{crit}$ should be less than 0.593 %. According to the simulation results, it indicates that the maximum strain 0.230 % of this oil pipeline does not exceed the allowable value.

### Displacement of pipelines

**Fig. 7:**
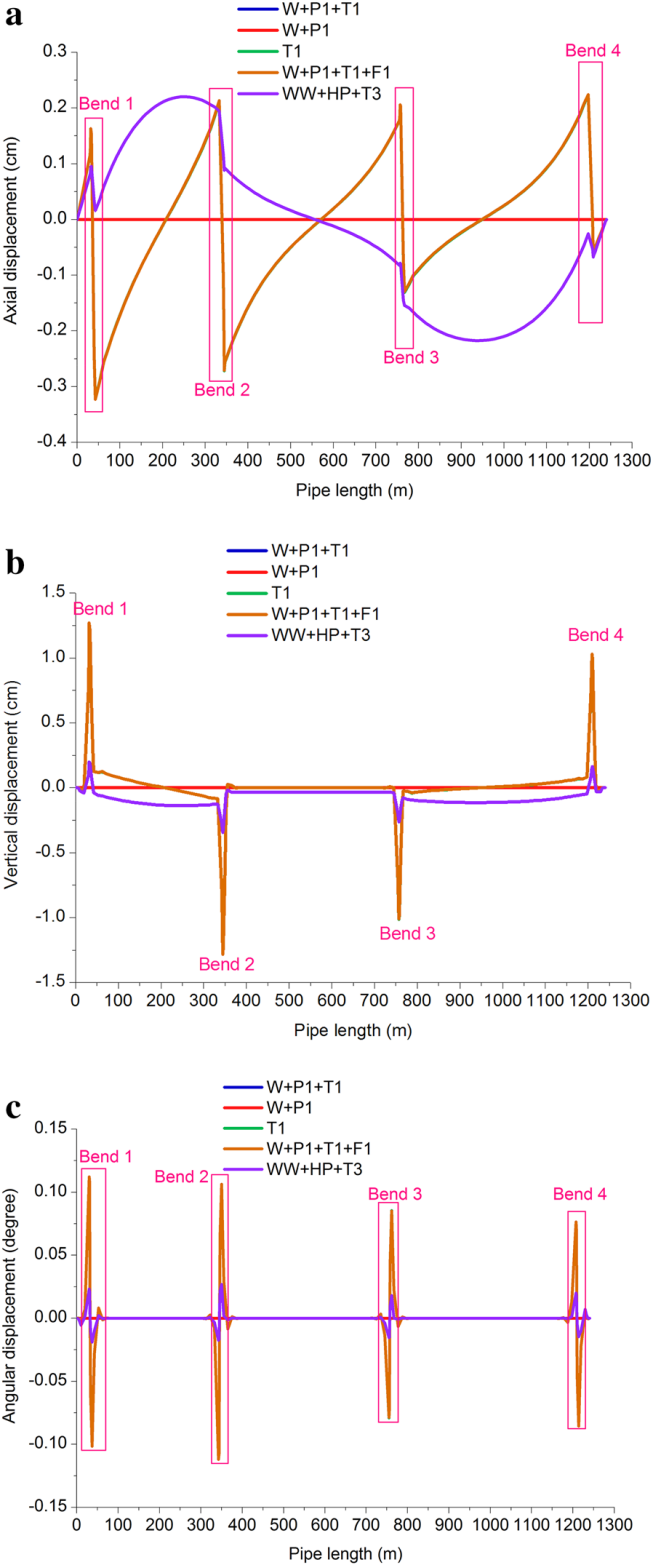
Displacement of gas pipeline: **a** axial displacement; **b** vertical displacement; and **c** Angular displacement

**Fig. 8:**
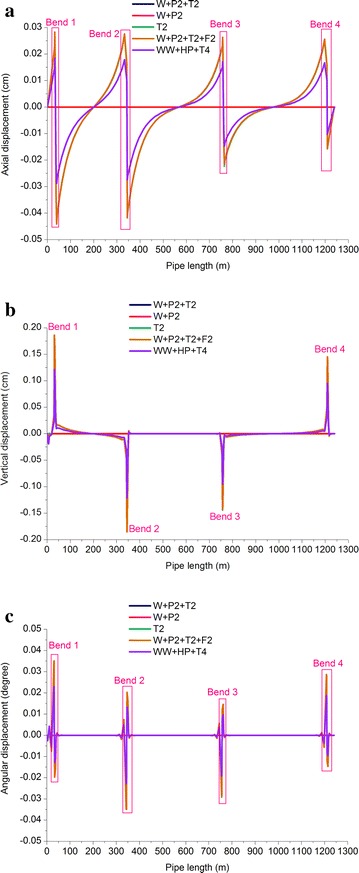
Displacement of oil pipeline: **a** axial displacement; **b** Vertical displacement; and **c** Angular displacement

Figures 7 and 8 illustrate the distributions of the axial displacement, vertical displacement, and angular displacement of the gas and oil pipelines (horizontal displacement was 0). Tables 7 and 8 show the checking of the maximum displacement (linear and angular displacements) of the oil and gas pipelines. It was observed that:

**Table 7:** Checking of the maximum displacement of gas pipeline

Loading conditions	Maximum axial displacement		Maximum vertical linear displacement		Maximum angular displacement	
	Absolute value (mm)	Location	Absolute value (mm)	Location	Absolute value (°)	Location
Operating case of gas pipeline	3.2	Bend 1	12.8	Bend 2	0.11	Bend 1
Sustained case of gas pipeline	0	–	0	–	0	–
Expansion case of gas pipeline	3.2	Bend 1	12.8	Bend 2	0.11	Bend 1
Pigging case of gas pipeline	3.2	Bend 1	12.8	Bend 2	0.11	Bend 1
Pressure test case of gas pipeline	2.21	Anchor block 1	3.46	Bend 2	0.03	Bend 2

**Table 8:** Checking of the maximum displacement of oil pipeline

Loading conditions	Maximum axial displacement		Maximum vertical linear displacement		Maximum angular displacement	
	Absolute value (mm)	Location	Absolute value (mm)	Location	Absolute value (°)	Location
Operating case of oil pipeline	0.44	Bend 1	1.85	Bend 1	0.03	Bend 1
Sustained case of oil pipeline	0	–	0.06	Fixed pier 1	0.001	Fixed pier 1
Expansion case of oil pipeline	0.44	Bend 1	1.85	Bend 1	0.03	Bend 1
Pigging case of oil pipeline	0.44	Bend 1	1.87	Bend 1	0.04	Bend 1
Pressure test case of oil pipeline	0.29	Bend 1	1.22	Bend 1	0.02	Bend 1

1. The pipe bracket of the buttress was 1.5 m and the maximum displacement of the horizontal pipeline was 12.8 mm, which was far smaller than 1500 × 40 % = 600 mm. The maximum angular displacement of the pipeline was generated at Bend 1 (gas pipeline) and was 0.11°, which was smaller than 4°, indicating that the section of parallel oil and gas pipelines met displacement requirements.
2. The linear and angular displacements of the gas pipeline were higher than those of the oil pipeline. In displacement control in practical engineering, the displacement of the gas pipeline should take priority.
3. The vertical displacements of the oil and gas pipelines were generally higher than the axial displacements. When pipelines are laid through tunnels, control of their vertical foundation subsidence should bear importance.
4. The displacements (linear and angular) of the gas and oil pipelines in the operating, expansion, and pigging tests were the highest and nearly equal, followed by those in the pressure test, while the displacements in the sustained test were the smallest.
5. The displacements of the gas and oil pipelines showed sudden changes at the bends, which was caused by deformation due to great flexibility of the pipes and the lack of supports.

## Discussion

The primary factors influencing stress in pipelines include the angle of inclined pipelines, the length of inclined pipelines, buttress interval and earthquake action. In addition, it is required to study stress reducing measures.

### Buttress interval

**Fig. 9:**
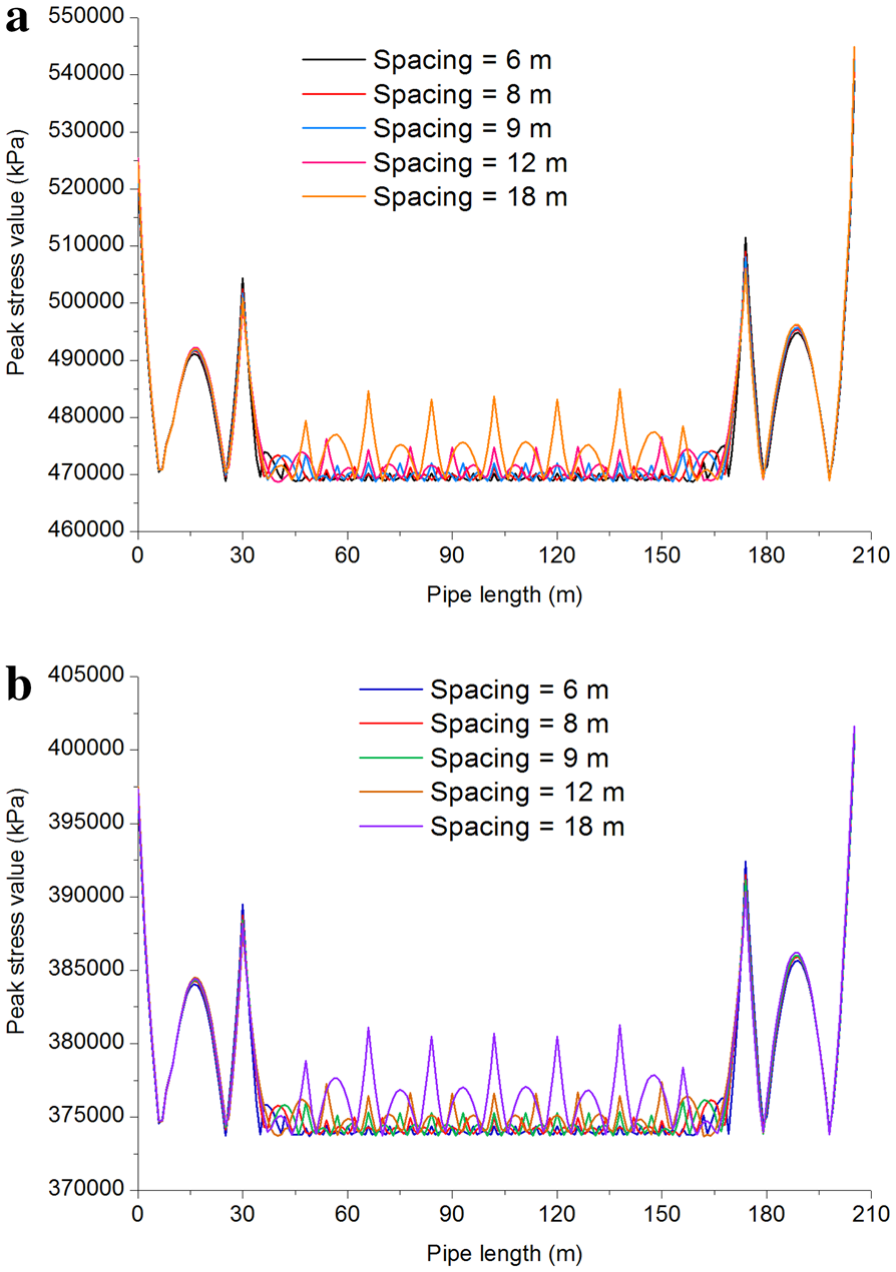
Peak stress distributions of the pipelines for different buttress intervals: **a** Oil pipeline; and **b** Gas pipeline

The buttress interval was analyzed for its influence on the average stress in a pipeline. In order to exclude the effect of the bends on the stress of pipelines, a model of a horizontal straight pipeline was established measuring 204 m long with fixed buttresses supporting both ends. The buttress interval was set as 6, 8, 9, 12, and 18 m for respective stress analysis of the gas and oil pipelines. The pressure test was selected as the research case (the temperature was 15 °C, the pressure in the gas pipeline was 15 MPa, the pressure in the oil pipeline was 13.5 MPa, and the medium in the pipelines was water) for the implementation of a limit state design. The peak stress distributions are shown in Fig. 9, and the analysis results of the average peak stress are shown in Table 9.

**Table 9:** Average peak stress of gas and oil pipelines

Pipelines	Spacing = 6 m	Spacing = 8 m	Spacing = 9 m	Spacing = 12 m	Spacing = 18 m
Gas pipeline	376.70 MPa	376.94 MPa	377.04 MPa	377.35 MPa	378.10 MPa
Oil pipeline	475.29 MPa	475.77 MPa	475.97 MPa	476.62 MPa	478.19 MPa

As indicated in Fig. 9 and Table 9, the average peak stress increased gradually as the buttress interval increased; the stress distributions of both the oil pipeline and the gas pipeline differed insignificantly, especially when the buttress interval changed from 6 to 12 m. Considering economic and construction interests, a buttress interval of 12 m is advised.

### Angle of the inclined pipelines

**Fig. 10:**
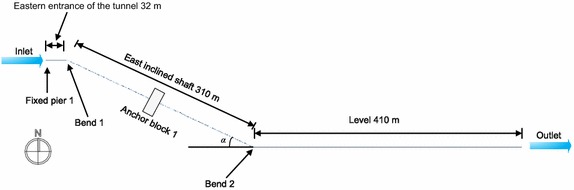
Basic pipeline model for different inclined angles

**Fig. 11:**
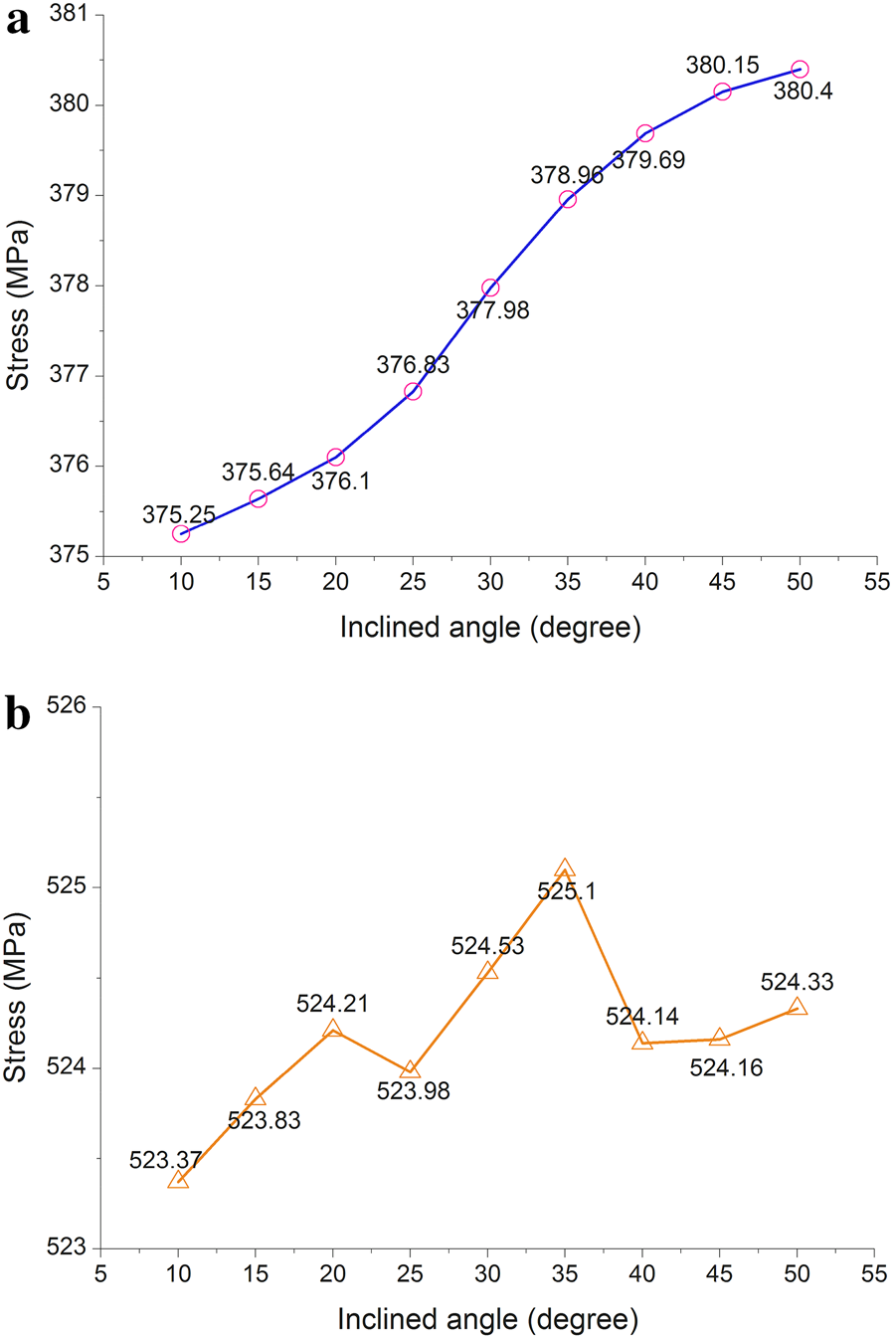
Maximum stress in the pipelines for different inclined angles under pressure test condition: **a** gas pipeline and **b** oil pipeline

A study was conducted on the angle of the inclined pipelines to analyze the stress at the bends. Due to the symmetrical structure of a tunnel, it was halved for analysis. The pipeline model was approximately 750 m long (Fig. 10), the range of the angle *α* was set as 10°–50°, and the stress was calculated every 5°. The change in the angle not only took into account the pressure condition in order to implement a limit state design. The maximum stress in the pipelines in the pressure test condition is shown in Fig. 11.

As indicated in Fig. 11, within an angle range of the inclined pipelines of 10°–50°, the maximum stress in the gas pipeline in the pressure tests, the maximum stress in the oil and gas pipelines showed no significant changes. In the angle range of 20°–35°, the oil pipeline’s maximum stress first increased and then decreased because in this range, the bending moment first decreased and then increased. Taking economic interests and the two pipelines into consideration, the angle should be no greater than 25°.

### Length of the inclined pipelines

**Fig. 12:**
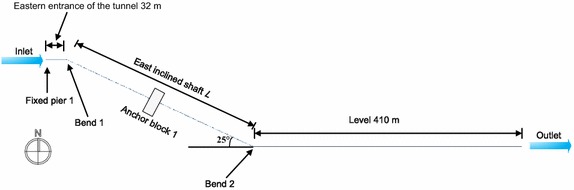
Basic research model for different lengths of inclined pipelines

Considering a limit state design, a study was conducted on the effects of the length of inclined pipelines on the stress (Fig. 12), and the pressure test condition was determined as the research case. The length of the inclined pipelines was increased from 10 m to study the pressure test condition.

**Fig. 13:**
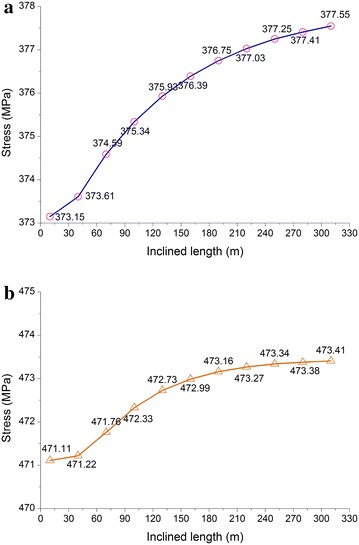
Stress in the pipelines for different lengths of inclined pipelines under pressure testing condition: **a** gas pipeline; and **b** oil pipeline

As illustrated in Fig. 13, the maximum stress in the pipelines changed slightly as the length of the inclined pipelines increased. In the design phase, because the determination of the length of the inclined parallel oil and gas pipelines should take into consideration the two pipelines, importance should be attached to checking the maximum stress in the oil pipeline in pressure test condition.

### Earthquake action

The project locates at earthquake fault zone. According to the directions of pipeline, we divided the directions of earthquake into: axial, vertical, horizontal and comprehensive earthquake action (Wu et al. 2015). On the basis of the data from Table 5.1.4-1 of GB 50011 (2010) *Code for seismic design of buildings*, the maximum value of horizontal seismic impact factor there is 0.90 g, and the axial, vertical and horizontal seismic acceleration of the pipeline are 0.29, 0.129 and 0.29 g, respectively.

We inputted the axial, vertical and horizontal seismic acceleration into CAESAR II software and established correspondence loading conditions:

- Seismic action condition in axial direction: W + P + T + U_*x*_
- Seismic action condition in vertical direction: W + P + T + U_*y*_
- Seismic action condition in horizontal direction: W + P + T + U_*z*_
- Seismic action condition in comprehensive direction: W + P + T + U_*x*_ + U_*y*_ + U_*z*_

Through the analysis of stress and displacement of oil and gas pipelines under seismic action and compared with the normal operating condition (W + P + T), main conclusions about overhead pipeline in tunnel can be obtained:

1. Under earthquake action, the maximum peak stress of gas pipeline is 492.61 MPa, the maximum peak stress of oil pipeline is 498.71 MPa. While under normal operation condition, the maximum peak stress of gas pipeline and oil pipeline are 362.21 MPa and 321.75 MPa respectively, which explains under earthquake action, the stress of oil pipelines enhances more obviously than that of gas pipelines.
2. Under earthquake action, the maximum axial displacement of gas pipeline is 7.75 mm, the maximum vertical displacement is 27.87 mm. The maximum axial displacement of oil pipeline is 1.03 mm, the maximum vertical displacement is 4.02 mm, indicating the displacement of gas pipeline under earthquake action is higher than that of oil pipeline. The vertical displacement of the pipeline is the largest and is followed by the axial displacement and then horizontal displacement. Analysis shows that, for pipelines in the inclined tunnel under earthquake action, the validation of the vertical displacement requires special attention and at the same time, relevant displacement restrictions should be added.

### Stress reducing measures: “ladder” laying

**Fig. 14:**
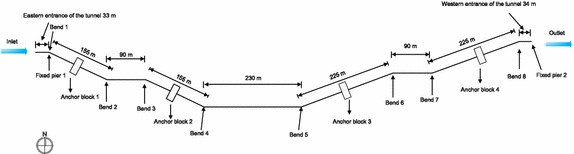
Schematic of pipeline with “Ladder” laying method

Based on conclusion in section “Angle of the inclined pipelines”: inclined pipe laying should try to reduce the inclination angle. However, when the crossing distance is constant (which means the crossing angle can’t be reduced), we put forward the way of the ladder laying. By increasing the number of pipe bend, we can reduce the overall stress of the pipe. In order to verify the correctness of this method and based on Fig. 4, we design ladder laying model, as shown in Fig. 14.

**Fig. 15:**
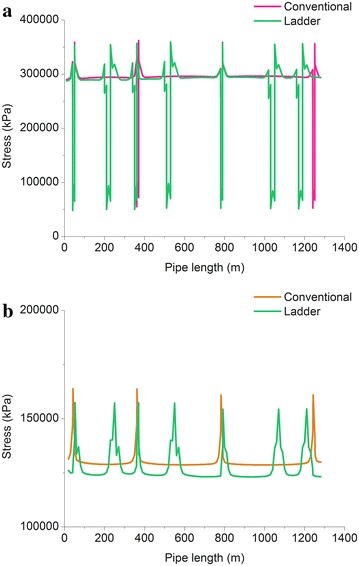
Stress distributions of the pipelines for different laying methods: **a** Gas pipeline; and **b** oil pipeline

Figure 15 displays stress distribution of oil and gas pipeline under different laying method and it can be seen that:

1. When using ladder laying method, pipe bend is still the position with higher stress. Due to the increase of pipe bend, the position where sudden change of stress appears is more than that using conventional laying method, but the stress of the bend is lower than that using the conventional laying method.
2. The average stress of gas pipeline which is by ladder laying is 275.87 MPa, while the conventional way of laying average stress is 285.57 MPa, and the decrease of the stress is about 3.40 %; The average stress of oil pipeline which is by ladder laying is 128.34 MPa, while the conventional way of laying average stress is 131.88 MPa, and the decrease of the stress is about 2.68 %.

Thus we can conclude that adopting the ladder laying method can reduce the overall stress of oil and gas pipelines. And the more the step, the lower the average stress.

### Angle of the tunnel entrance and exit guide

**Fig. 16:**
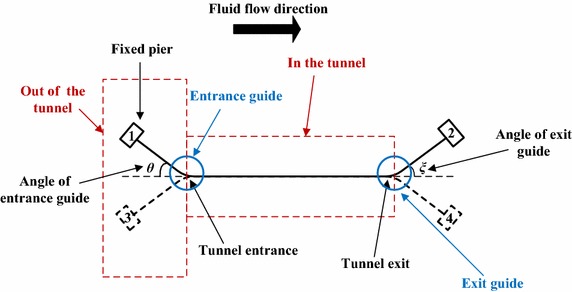
Tunnel entrance and exit guide plane sketch

Due to the tunnel entrance and exit are fixed constraint, displacement of tunnel pipe is completely limited, leading to stress concentration at local site (pipe bend). Therefore, consider using horizontal pipe bend with a certain angle in tunnel entrance and exit, as indicated in Fig. 16.

Guide of tunnel entrance and exit is horizontal pipe bend, as shown in Fig. 16. There are 4 kinds of entrance and exit guide direction resettlement way: 1–2, 1–4, 2–3, 3–4. After the study, it is found that the combinations of these four ways have little influence on stress, so we only discuss the angle of tunnel entrance and exit guide.

Taking resettlement way 1–2 as example, the pipe model of θ = ζ = 15°, 30°, 45°, 60° are analyzed. We summarize the stress value at Bend 1, Bend 2, Bend 3 and Bend 4, as shown in Tables 10, 11.

**Table 10:** Different tunnel entrance and exit guide angle corresponding to the stress of four bend (Gas pipeline)

Location	No entrance and exit guides	Guide angle = 15°	Guide angle = 30°	Guide angle = 45°	Guide angle = 60°
Bend 1	358.92 MPa	358.62 MPa	351.91 MPa	343.62 MPa	334.83 MPa
Bend 2	362.24 MPa	362.19 MPa	361.74 MPa	361.15 MPa	360.55 MPa
Bend 3	358.76 MPa	358.76 MPa	358.47 MPa	358.12 MPa	357.77 MPa
Bend 4	356.13 MPa	354.35 MPa	342.23 MPa	327.34 MPa	312.00 MPa

**Table 11:** Different tunnel entrance and exit guide angle corresponding to the stress of four bend (Oil pipeline)

Location	No entrance and exit guides	Guide angle = 15°	Guide angle = 30°	Guide angle = 45°	Guide angle = 60°
Bend 1	411.39 MPa	327.91 MPa	327.43 MPa	326.95 MPa	326.47 MPa
Bend 2	412.65 MPa	328.21 MPa	328.21 MPa	328.17 MPa	328.17 MPa
Bend 3	421.69 MPa	329.77 MPa	329.77 MPa	329.77 MPa	329.77 MPa
Bend 4	358.70 MPa	329.12 MPa	327.78 MPa	326.52 MPa	325.34 MPa

From Tables 10, 11, it can be concluded:

1. For gas pipeline and the oil pipeline, adding guide bend at tunnel entrance and exit will help to reduce the stress of the bend in the tunnel, and especially for oil pipeline, stress reducing effect is obvious;
2. For gas pipeline, with the increase of guide bend angle, the stress at the bend declines, and the trend is obvious, suggesting that in the design of tunnel gas pipeline we should choose larger guide angle;
3. For oil pipeline, the entrance and exit guide has helped to reduce the stress in a large degree. But with the increase of guide bend angle, the stress reducing effect doesn’t change much. Therefore, it suggests that for oil pipeline, we only consider landscape outside the tunnel and construction convenience.

## Conclusions

Through stress analysis of a section of the parallel oil and gas pipelines, the locations of the critical sections and the main loads affecting stress of the gas and crude oil pipelines running through a tunnel were obtained.

The pressure test revealed a dangerous condition of the oil and gas pipelines. In most cases, the stress at the bends (Bend 2 and Bend 3) at the bottom of the tunnel was the greatest, and was caused by the lack of supports at the bends. It was also discovered that the effects of the gravity of the fluid were greater in the pipelines at Bends 2 and 3 than at Bends 1 and 4.

The displacements of the gas and oil pipelines changed suddenly at the bends, which was caused by the deformation due to the flexibility of the pipes and the lack of supports at the bends. The linear and angular displacements of the gas pipeline were higher than those of the oil pipeline. Displacement control in practical engineering should put considerable emphasis on checking the displacement of the gas pipeline.

Analysis of the factors influencing stress concluded that: (1) The buttress interval be 12 m considering economic and construction interests; (2) The angle of the inclined pipelines should be no greater than 25° based on the overall consideration of economy, the two pipelines; (3) The determination of the length of the inclined parallel oil and gas pipelines took the two pipelines into consideration, and importance should be attached to checking the maximum stress in the oil pipeline in the pressure conditions; (4) Under earthquake action, the stress of oil pipelines enhances more obviously than that of gas pipelines; (5) The vertical displacements of the pipelines is the largest under earthquake action; (6) The average stress can be reduced by adopting “ladder” laying; (7) Guide bend can be set at the tunnel entrance and exit in order to reduce the stress. And oil pipeline stress reducing effect is obvious. For gas pipeline, we should choose guide bend with lager angle, while for oil pipeline, we only consider landscape outside the tunnel and construction convenience.
